# Triglycerides and risk of cardiovascular events in statin-treated patients with newly diagnosed type 2 diabetes: a Danish cohort study

**DOI:** 10.1186/s12933-023-01921-5

**Published:** 2023-07-26

**Authors:** Frederik Pagh Bredahl Kristensen, Diana Hedevang Christensen, Martin Bødtker Mortensen, Michael Maeng, Johnny Kahlert, Henrik Toft Sørensen, Reimar Wernich Thomsen

**Affiliations:** 1 Department of Clinical Epidemiology, Aarhus University Hospital and Aarhus University, Olof Palmes Alle 43-45, 8200 Aarhus N, Denmark; 2 Department of Endocrinology and Internal Medicine, Aarhus University Hospital, Aarhus, Denmark; 3 Department of Cardiology, Aarhus University Hospital, Aarhus N, Denmark; 4 Ciccarone Center for the Prevention of Cardiovascular Disease, Johns Hopkins University School of Medicine, Baltimore, USA

**Keywords:** Type 2 diabetes, Residual cardiovascular risk, Statin-treated patients, Triglyceride-rich lipoproteins, Remnant cholesterol, Routine clinical care

## Abstract

**Background:**

Elevated triglyceride levels are a clinically useful marker of remnant cholesterol. It is unknown whether triglycerides are associated with residual cardiovascular risk in CVD-naïve patients with newly diagnosed type 2 diabetes mellitus (T2DM), who are already on statin therapy. We aimed to assess the association between triglyceride levels and risk of major cardiovascular events (MACE) in statin-treated patients with newly diagnosed T2DM managed in routine clinical care.

**Methods:**

This cohort study included newly diagnosed T2DM patients without a previous diagnosis of cardiovascular disease in Northern Denmark during 2005–2017. Individual triglyceride levels while on statin treatment were assessed within 1 year after T2DM diagnosis. The primary outcome was a composite of myocardial infarction, ischemic stroke, or cardiac death (MACE). Patients were followed from one year after T2DM diagnosis until 30 April 2021, MACE, emigration, or death. We used Cox regression to compute hazard ratios (HRs) controlling for confounding factors.

**Results:**

Among 27,080 statin-treated patients with T2DM (median age 63 years; 53% males), triglyceride levels were < 1.0 mmol/L in 17%, 1.0–1.9 mmol/L in 52%, 2.0–2.9 mmol/L in 20%, and ≥ 3.0 mmol/L in 11%. During follow-up, 1,957 incident MACE events occurred (11.0 per 1000 person-years). Compared with triglyceride levels < 1.0 mmol/L, confounder-adjusted HRs for incident MACE were 1.14 (95% CI 1.00–1.29) for levels between 1.0 and 1.9 mmol/L, 1.30 (95% CI 1.12–1.51) for levels between 2.0 and 2.9 mmol/L, and 1.44 (95% CI 1.20–1.73) for levels ≥ 3.0 mmol/L. This association was primarily driven by higher rates of myocardial infarction and cardiac death and attenuated only slightly after additional adjustment for LDL cholesterol. Spline analyses confirmed a linearly increasing risk of MACE with higher triglyceride levels. Stratified analyses showed that the associations between triglyceride levels and MACE were stronger among women.

**Conclusions:**

In statin-treated patients with newly diagnosed T2DM, triglyceride levels are associated with MACE already from 1.0 mmol/L. This suggests that high triglyceride levels are a predictor of residual cardiovascular risk in early T2DM and could be used to guide allocation of additional lipid-lowering therapies for CVD prevention.

**Supplementary Information:**

The online version contains supplementary material available at 10.1186/s12933-023-01921-5.

## Introduction

Low-density lipoprotein (LDL) cholesterol has been the primary lipid target for cardiovascular disease (CVD) prevention for decades [1]. However, triglyceride-rich lipoproteins, including very low-density and intermediate-density lipoproteins, also play a role in CVD development [2–4]. In addition to triglycerides, these lipoproteins carry remnant cholesterol that contributes to development of atherosclerosis [2–7].

Elevated triglycerides are a clinically useful marker of remnant cholesterol [8]. High triglyceride levels are found in patients with type 2 diabetes mellitus (T2DM) as a component of the metabolic syndrome, together with low HDL cholesterol, obesity, and hypertension [9–13]. High triglyceride levels have been linked to increased risk of CVD among patients with long-standing T2DM or prevalent CVD [10, 14–19]. In contrast, a gap exists in our understanding of how triglyceride levels impact CVD risk among *CVD-naïve patients with newly diagnosed T2DM who are already treated with statins*. These patients are early in their course of disease where preventive initiatives may be most effective [20, 21].

Lifestyle interventions and statin therapy are key components in lowering LDL cholesterol and triglyceride levels and annual lipid assessment is recommended for T2DM patients [22]. While no target is specified for triglycerides, T2DM patients without known CVD should aim for a LDL cholesterol level < 2.6 mmol/L [22]. To improve clinical guidance for determining which patients to treat with additional preventive medications in contemporary practice, large cohort studies are needed to investigate the association between triglyceride levels and CVD risk in T2DM patients already treated with statin therapy, where a residual CVD risk may exist [1, 2, 21, 22].

We therefore conducted a cohort study of statin-treated, newly diagnosed T2DM patients without known CVD. We examined whether elevated triglyceride levels, measured within one year after T2DM diagnosis, were associated with increased risk of major adverse cardiovascular events (MACE) in a population-based setting.

## Methods

### Setting and design

This cohort study used existing medical databases covering the entire population of the Northern Denmark [23, 24]. We linked individual-level data from routine clinical care and administrative databases using the central personal registration number assigned to each Danish resident at birth and upon migration, allowing lifelong follow-up [23]. In Denmark, most T2DM patients (~ 80%) are diagnosed and followed in general practice. The remaining patients, *e.g.,* those with severe complications, receive specialist care in hospital outpatient clinics [25]. A description of the used databases is provided in Additional file 1: Table S1.

This study was approved by the Danish Health Data Authority and registered at Aarhus University on behalf of the Danish Data Protection Agency (No. 2016-051-000001/438/812). According to Danish legislation, ethical committee approval is not needed for register-based studies.

### Study cohort

**Fig. 1:**
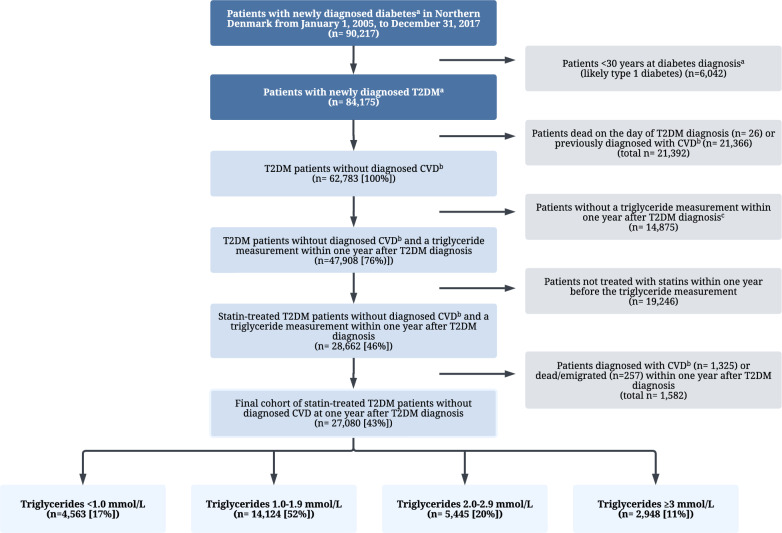
Flowchart of inclusion in the T2DM study cohort. We used 1 mmol/L cut-offs to categorize patients. ^a^Newly diagnosed diabetes was defined as either 1) a first-time hospital inpatient or outpatient clinic diagnosis of diabetes or 2) a first-time redeemed prescription for a glucose-lowering drug issued by either a primary care or a hospital-based physician. ^b^Cardiovascular disease was defined as a hospital recorded diagnoses of myocardial infarction, angina pectoris, heart failure, ischemic stroke, hemorrhagic stroke, or peripheral arterial disease or a CVD-related procedure including PCI/CABG, thrombolysis/thrombectomy, or lower limb revascularization/amputation. ^c^4348 (29%) were statin users and potentially eligible in our study if they had had a triglyceride measurement. Please see Additional file 1: Table S8 for a baseline characterization of these patients. CVD, cardiovascular disease; PCI/CABG, percutaneous coronary intervention/ coronary artery bypass graft; T2DM, type 2 diabetes mellitus

The sampling of the cohort is described in Fig. 1 and Additional file 1: Fig. S1. We used the Danish National Patient Registry and the Danish National Prescription Registry to identify all patients with newly diagnosed T2DM who resided in Northern Denmark between January 1, 2005 and December 31, 2017. Patients with newly diagnosed T2DM had either a *first-time* hospital recorded inpatient or outpatient diagnosis of diabetes or a *first-time* redemption of a glucose-lowering drug (GLD) prescription issued by a primary care physician or a hospital-based physician. We excluded patients who were younger than 30 years at diabetes diagnosis to minimize inclusion of type 1 diabetes. We further excluded patients diagnosed with CVD (myocardial infarction, angina pectoris, heart failure, stroke, peripheral artery disease or undergone coronary/limb revascularization/amputation, thrombolysis/thrombectomy) any time before and up to one year after a T2DM diagnosis, patients without any triglyceride measurement in the year after T2DM diagnosis, and patients who had not used statins within one year prior to the latest triglyceride measurement (See definitions in Additional file 1: Table S1). Follow-up began one year after the T2DM diagnosis date (index date) (Additional file 1: Fig. S1).

### Assessment of triglycerides

Non-fasting triglyceride levels were measured using standard laboratory assays performed during routine clinical care and recorded in the Clinical Laboratory Information System (LABKA) [24]. LABKA has collected the results of routinely measured blood tests performed in the population of Northern Denmark, in both general practice and hospital settings, since the late 1990s [24]. We obtained the latest measured triglyceride value within one year after T2DM diagnosis. This allowed time for initial diagnostics, lifestyle modifications and lipid-lowering therapy, and initial follow-up visits as a new T2DM patient either in general practice or at a hospital clinic according to Danish T2DM guidelines. If more than one triglyceride measurement was available, we used the latest measured value before the index date (one year after T2DM diagnosis). We then grouped patients according to their triglyceride level (< 1.0 mmol/L, 1.0–1.9 mmol/L, 2.0–2.9 mmol/L, and 3.0 mmol/L) to reflect the CVD risk for each 1.0 mmol/L increase in triglyceride levels, as previously described [3, 5, 20].

### Endpoints

The primary endpoint was a composite of non-fatal acute myocardial infarction, non-fatal ischemic or unspecified stroke (ischemic stroke), and cardiac death (MACE). We ascertained endpoints from the Danish National Patient Registry and the Danish Register of Causes of Death, using all available primary inpatient discharge diagnoses of myocardial infarction and all primary or secondary inpatient discharge diagnoses of ischemic stroke. Validation studies have previously reported a positive predictive value (PPV) above 90% for diagnoses of myocardial infarction and between 70 and 95% for ischemic stroke diagnoses in the Danish National Patient Registry [26, 27].

### Other covariates

We collected data from the above registries on a wide range of factors potentially associated with both triglyceride levels and risk of future CVD. These included age on the index date, biological sex, markers of lifestyle (smoking, alcohol abuse), diabetes stage (use of glucose-lowering drugs, HbA1c), metabolic risk factors (hospital-diagnosed obesity, hypertension, total cholesterol, high-density lipoprotein [HDL] cholesterol, LDL cholesterol), kidney function (eGFR), comorbidities and diabetic complications (atrial fibrillation, cancer, chronic liver disease, diabetic eye disease, diabetic kidney disease, use of cardiovascular drugs), and psychiatric comorbidity (use of antidepressant or antipsychotic drugs). As exact information on the degree of alcohol overuse, obesity, hypertension, and smoking is not recorded in the registries, we used diagnosis codes and medication prescriptions to create proxy markers for these variables (Additional file 1: Table S1). LDL cholesterol was derived from Friedewald’s formula, and non-HDL cholesterol was calculated as total cholesterol minus HDL cholesterol. The definition of each covariate is presented in Additional file 1: Table S1.

### Statistical analyses

We described baseline characteristics of all patients according to triglyceride level on their index date. Patients were followed from their index date until occurrence of incident MACE, non-CVD death, emigration, or administrative end of follow-up (December 31, 2019), whichever came first. In secondary analyses, we calculated follow-up times and incidence rates separately for myocardial infarction, ischemic stroke, and cardiac death. Cox proportional hazard regression models were used to calculate adjusted hazard ratios (HRs), using patients with triglyceride levels < 1.0 mmol/L as the reference group. The main model was chosen prior to data analysis and adjusted for covariates that—based on previous knowledge—likely associated with triglyceride levels and MACE endpoints [1, 22], including age, sex, calendar year, smoking, hypertension, eGFR, use of GLDs including insulin, and HbA1c level. We did not adjust for HDL cholesterol due to the non-causal association with CVD and high correlation with triglyceride levels [2, 4, 22, 28, 29]. The main analyses were conducted on the whole study population, but also in men and women separately. Missing data on covariates (varying from 1% to 3%) were imputed using multiple chained equations, assuming that data were missing at random (See Methods S1 on missing values and data imputation in Additional file 1: Methods S1) [30]. On a continuous scale, restricted cubic spline models with four degrees of freedom were used to examine associations between triglyceride levels, MACE, and each outcome [31]. Knots were placed at default locations [31]. Examination of Schoenfeld residuals and log-minus-log plots indicated that no deviations from the proportionality of hazard assumption occurred in any analysis.

### Additional analyses

We performed seven additional analyses to examine the robustness of the associations:

1. An extended adjusted analysis, additionally including obesity, cancer, alcohol abuse, chronic liver disease, psychiatric comorbidity, and aspirin use.
2. An analysis with additional adjustment for LDL cholesterol, to examine the potential residual CVD risk, beyond the effect of LDL cholesterol. This analysis was restricted to patients with triglyceride levels < 4.0 mmol/L to provide a valid estimate of LDL cholesterol derived from Friedewald’s formula (N = 26,502, 98%) [22, 32].
3. An analysis restricted to patients with triglycerides measured within six months after diagnosis of T2DM (n = 19,980, 74%), starting follow-up at six months to capture early MACE events.
4. An analysis applying a common dichotomous cut-off value for hypertriglyceridemia (≥ 1.7 mmol/L]), permitting direct comparison with previous studies [21], and another analysis using clinical guideline cut-offs values (< 1.0 mmol/L, 1.0–1.69 mmol/L, 1.7–2.29 mmol/L, ≥ 2.3 mmol/L) [21, 22].
5. An analysis investigating the effect measure modification of prior statin treatment duration.
6. A baseline characterization of the patients that would have been included in our study population *if* they had had a triglyceride measurement (N = 4,348, please see Fig. 1).
7. An analysis calculating E-values, assessing the minimum strength needed by any unmeasured confounder to fully explain the observed associations [33].

We used Stata (version 17.0, Statacorp) for all data management, statistical analyses, and graphical illustrations.

## Results

### Triglyceride levels and patient characteristics on the index date

Among 62,783 (100%) T2DM patients without known CVD, 47,908 (76%) had a triglyceride measurement within one year after diagnosis and 28,662 (46%) were statin users. After exclusion of patients who died, emigrated, or had a CVD event within one year after T2DM diagnosis, the final analytic cohort included 27,080 (43%) patients (Fig. 1).

The median triglyceride level achieved on statin therapy within one year after T2DM diagnosis was 1.5 mmol/L (quartiles: 1.1, 2.1 mmol/L), based on the latest available triglyceride value measured a median of 89 days (quartiles: 184, 327 days) before the index date. Compared with patients with triglyceride levels < 1.0 mmol/L, patients with triglyceride levels ≥ 3.0 mmol/L were more often male (69% *vs.* 56%) were on average 10 years younger (median age: 55 years *vs.* 65 years), and had higher non-HDL cholesterol levels (median value 3.7 mmol/L *vs.* 2.2 mmol/L). In contrast, they had similar HbA1c values (median 49 mmol/mol *vs*. 45 mmol/mol) while receiving more intensive GLD therapy (≥ 1 GLD or insulin therapy: 27% *vs.* 17%) (Table 1 and Additional file 1: Table S2).

**Table 1:** Characteristics of 27,080 statin-treated patients by triglyceride level measured up to one year after T2DM diagnosis

Triglyceride level (mmol/L)	< 1.0	1.0–1.9	2.0–2.9	≥ 3.0
N	4563 (17%)	14,124 (52%)	5445 (20%)	2948 (11%)
Male	2538 (56%)	7196 (51%)	2860 (53%)	1826 (62%)
Median age (quartiles), years	65 (56–72)	64 (55–71)	61 (52–69)	56 (49–65)
Calendar year				
2005–2008	789 (17%)	2379 (17%)	824 (15%)	459 (16%)
2009–2012	1955 (43%)	5574 (39%)	1970 (36%)	961 (33%)
2013–2016	1341 (29%)	4191 (30%)	1743 (32%)	971 (33%)
2017–2018	478 (10%)	1980 (14%)	908 (17%)	557 (19%)
Days from latest triglyceride measurement until start of follow-up (quartiles)	91 (42–182)	91 (40–183)	87 (37–179)	84 (36–176)
Smoking	444 (10%)	1474 (10%)	660 (12%)	342 (12%)
Alcohol abuse	131 (3%)	314 (2%)	153 (3%)	112 (4%)
Obesity	220 (5%)	1193 (8%)	633 (12%)	320 (11%)
Hypertension	1928 (42%)	6635 (47%)	2636 (48%)	1347 (46%)
Median HbA1c, % (quartiles)	6.3 (5.9–6.6)	6.4 (6.0–6.8)	6.5 (6.1–6.9)	6.6 (6.2–7.3)
Median HbA1c, mmol/mol (quartiles)	45 (41–48)	46 (42–51)	48 (43–52)	49 (44–56)
Median total cholesterol, mmol/L (quartiles)	3.8 (3.3–4.3)	4.0 (3.5–4.5)	4.3 (3.8–4.9)	4.8 (4.2–5.5)
Median LDL cholesterol, mmol/L (quartiles)	1.8 (1.5–2.2)	2.0 (1.7–2.5)	2.1 (1.7–2.7)	2.1 (1.6–2.8)
Median HDL cholesterol, mmol/L (quartiles)	1.5 (1.3–1.8)	1.3 (1.1–1.5)	1.1 (1.0–1.3)	1.0 (0.8–1.2)
Median non-HDL cholesterol, mmol/L (quartiles)	2.2 (1.9–2.6)	2.6 (2.3–3.1)	3.1 (2.7–3.7)	3.7 (3.1–4.5)
Median eGFR, mL/min/1.73m2 (quartiles)	89 (78–98)	89 (76–98)	90 (76–100)	95 (80–104)
Comorbidities (n, %)				
Diabetic eye complications	160 (4%)	480 (3%)	142 (3%)	76 (3%)
Diabetic kidney complications	39 (1%)	151 (1%)	96 (2%)	63 (2%)
Atrial fibrillation	173 (4%)	605 (4%)	276 (5%)	117 (4%)
Chronic liver disease	49 (1%)	144 (1%)	64 (1%)	44 (1%)
Any cancer	315 (7%)	1,048 (7%)	399 (7%)	181 (6%)
Identification of diabetes patients				
GLD prescription	3811 (84%)	12,206 (86%)	4702 (86%)	2,536 (86%)
Diabetes diagnosis code	752 (16%)	1918 (14%)	743 (14%)	412 (14%)
Medication use (n, %)				
Non-insulin GLD monotherapy	3789 (83%)	11,829 (84%)	4411 (81%)	2222 (75%)
Insulin or GLD polytherapy	774 (17%)	2295 (16%)	1034 (19%)	726 (25%)
Insulin	291 (6%)	519 (4%)	227 (4%)	170 (6%)
Aspirin	1344 (29%)	3914 (28%)	1421 (26%)	671 (23%)
Anticoagulants	264 (6%)	992 (7%)	438 (8%)	198 (7%)
Loop diuretics	259 (6%)	1205 (9%)	596 (11%)	330 (11%)
Non-loop diuretics	946 (21%)	3439 (24%)	1431 (26%)	632 (21%)
Renin-angiotensin-system antagonists	2688 (59%)	8800 (62%)	3374 (62%)	1,764 (60%)
Calcium channel antagonists	1191 (26%)	4035 (29%)	1456 (27%)	775 (26%)
Beta blockers	692 (15%)	2973 (21%)	1313 (24%)	739 (25%)
Antipsychotics and anticonvulsants	250 (5%)	1065 (8%)	616 (11%)	418 (14%)
Antidepressants including SSRIs	536 (12%)	2131 (15%)	1143 (21%)	726 (25%)

Data are numbers and percent unless otherwise specified. Missing data for laboratory values varied between 1 and 3% (Additional file 1: Table S2). See Additional file 1: Table S1 for definitions of covariates

*eGFR* glomerular filtration rate; *GLD* glucose-lowering drug; *HDL* high-density lipoprotein; *LDL* low-density lipoprotein; *SSRI* selective serotonin reuptake inhibitors; *T2DM* Type 2 diabetes mellitus

### Association of triglyceride levels with MACE

**Fig. 2:**
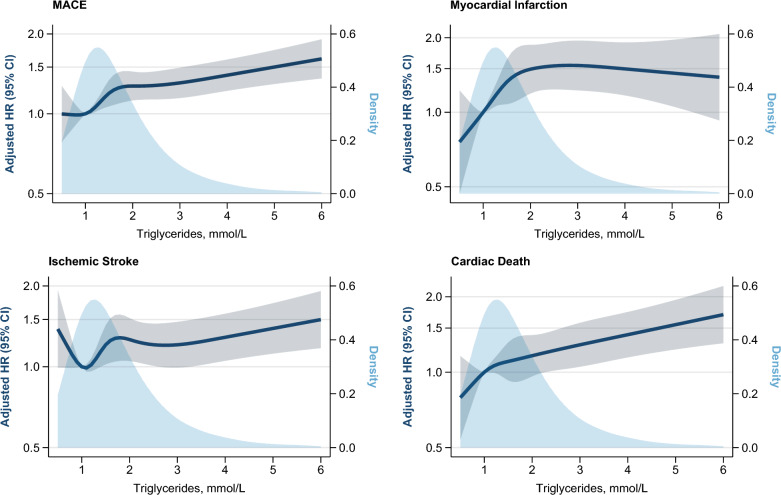
Adjusted hazard ratios of MACE, myocardial infarction, ischemic stroke, and cardiac death associated with triglyceride levels in statin-treated patients. Splines were analyzed in a subcohort of patients who had complete information on all covariates included in the model (n = 26,098 [96%]). We excluded outliers outside of the 1st and 99th percentile of triglyceride distribution (triglycerides = 0.5 mmol/L and 6.2 mmol/L [rounded to 6.0 mmol/L]). MACE was a composite outcome of acute myocardial infarction, ischemic stroke, and cardiac death within a median follow-up time of 6.7 years (quartiles: 3.6, 9.1 years). Solid navy lines are adjusted HRs and grey shades depict the 95% confidence interval based on the restricted cubic spline regression. The reference value was 1.0 mmol/L. The location of the knots was determined by the percentiles recommended in previous literature, corresponding to the 5th, 35th, 65th, and 95th percentiles [31, 43]. In the study cohort, the 5th percentile of the triglyceride distribution corresponded to 0.5 mmol/L and the 95th percentile corresponded to 3.8 mmol/L. The model was adjusted for age, sex, calendar year, markers of smoking, hypertension, kidney function (eGFR), glucose-lowering drug therapy (including insulin), and HbA1c levels. *CVD* cardiovascular disease; *HR* hazard ratio; *MACE* major adverse cardiovascular events

During median follow-up of 6.7 years (quartiles: 3.6, 9.1 years), 1,957 MACE events occurred (11.0 per 1000 person-years). Figure 2 shows restricted cubic spline models of the association between triglyceride levels and each outcome. Continuous values of triglycerides showed a linearly increasing adjusted HR for MACE and cardiac death. The slope for myocardial infarction showed a similar pattern but reached a plateau at triglyceride levels above 2.5 mmol/L. The slope for ischemic stroke was U-shaped and additionally showed a more moderately increasing adjusted HR for triglyceride levels above 2.5 mmol/L (Fig. 2).

Compared with patients with triglyceride levels < 1.0 mmol/L, the main model adjusted HRs for MACE were 1.14 (95% CI 1.00–1.29) for triglyceride levels between 1.0 and 1.9 mmol/L, 1.30 (95% CI 1.12–1.51) for patients with triglyceride levels between 2.0 and 2.9 mmol/L, and 1.44 (95% 1.20–1.73) for patients with triglyceride levels ≥ 3.0 mmol/L (Table 2). Associations between the three increasing triglyceride levels and MACE were driven in particular by higher HRs for myocardial infarction (HRs 1.35 [95% CI 1.06–1.71], 1.67 [95% CI 1.27–2.20], and 1.75 [95% CI 1.27–2.42]) and for cardiac death (HRs 1.20 [95% CI 0.99–1.46], 1.37 [95% CI 1.08–1.72], and 1.53 [95% CI 1.14–2.06]), while the three increasing triglyceride levels showed a weaker association with risk of ischemic stroke (HRs 0.96 [95% CI 0.81–1.15], 1.04 [95% CI 0.84–1.29], and 1.13 [95% CI 0.86–1.47]) (Table 2). The associations between triglyceride levels and MACE were stronger among women (Table 2).

**Table 2:** Adjusted hazard ratios for study outcomes associated with triglyceride levels in statin-treated patients

Triglyceride level (mmol/L)	Overall			Males			Females		
	N/events	Incidence rates per 1000 PY (95% CI)	Adjusted HR (95% CI)	N/events	Incidence rates per 1000 PY (95% CI)	Adjusted HR (95% CI)	N/events	Incidence rates per 1000 PY (95% CI)	Adjusted HR (95% CI)
Mace									
< 1.0	4563/322	10.2 (9.2–11.4)	1.0	2538/199	11.5 (10.0–13.2)	1.0	2025/123	8.7 (7.3–10.4)	1.0
1.0–1.9	14,124/1,051	11.2 (10.5–11.8)	1.14 (1.00–1.29)	7196/608	12.9 (11.9–13.9)	1.17 (0.99–1.37)	6928/443	9.4 (8.6–10.3)	1.09 (0.89–1.34)
2.0–2.9	5445/390	11.3 (10.3–12.5)	1.30 (1.12–1.51)	2860/208	11.5 (10.0–13.2)	1.18 (0.97–1.44)	2585/182	11.1 (9.6–12.9)	1.45 (1.15–1.82)
≥ 3.0	2948/194	10.6 (9.2–12.2)	1.44 (1.20–1.73)	1826/132	11.8 (9.9–14.0)	1.47 (1.17–1.85)	1122/62	8.8 (6.9–11.3)	1.32 (0.97–1.80)
Myocardial infarction									
< 1.0	4563/84	2.3 (1.8–2.8)	1.0	2538/61	3.0 (2.3–3.8)	1.0	2025/23	1.4 (0.9–2.1)	1.0
1.0–1.9	14,124/327	2.9 (2.6–3.3)	1.35 (1.06–1.71)	7196/202	3.6 (3.2–4.2)	1.22 (0.92–1.63)	6928/125	2.3 (1.9–2.7)	1.66 (1.07–2.60)
2.0–2.9	5445/141	3.5 (2.9–4.1)	1.67 (1.27–2.20)	2860/84	3.9 (3.2–4.9)	1.39 (1.00–1.95)	2585/57	2.9 (2.3–3.8)	2.39 (1.46–3.89)
≥ 3.0	2948/74	3.4 (2.7–4.3)	1.75 (1.27–2.42)	1826/57	4.2 (3.3–5.5)	1.65 (1.13–2.41)	1122/17	2.0 (1.3–3.3)	1.78 (0.94–3.37)
Ischemic stroke									
< 1.0	4563/172	4.7 (4.0–5.4)	1.0	2538/99	4.9 (4.0–5.9)	1.0	2025/73	4.4 (3.5–5.9)	1.0
1.0–1.9	14,124/483	4.4 (4.0–4.8)	0.96 (0.81–1.15)	7196/277	5.0 (4.4–5.6)	1.06 (0.84–1.33)	6928/206	3.8 (3.7–4.3)	0.83 (0.63–1.09)
2.0–2.9	5445/173	4.2 (3.7–4.9)	1.04 (0.84–1.29)	2860/93	4.3 (3.5–5.3)	1.04 (0.78–1.39)	2585/80	4.1 (3.3–5.1)	1.01 (0.73–1.39)
≥ 3.0	2948/88	4.0 (3.3–5.0)	1.13 (0.86–1.47)	1826/59	4.4 (3.4–5.7)	1.23 (0.87–1.72)	1122/29	3.5 (2.4–5.0)	0.97 (0.62–1.50)
Cardiac death									
< 1.0	4563/134	4.1 (3.5–4.9)	1.0	2538/86	4.8 (3.9–6.0)	1.0	2025/48	3.3 (2.5–4.4)	1.0
1.0–1.9	14,124/454	4.7 (4.3–5.2)	1.20 (0.99–1.46)	7196/254	5.2 (4.6–5.9)	1.17 (0.91–1.49)	6928/200	4.2 (3.6–4.8)	1.28 (0.94–1.76)
2.0–2.9	5445/161	4.6 (3.9–5.3)	1.37 (1.08–1.72)	2860/76	4.1 (3.3–5.1)	1.10 (0.80–1.50)	2585/85	5.1 (4.1–6.2)	1.79 (1.25–2.56)
≥ 3.0	2948/73	3.9 (3.1–4.9)	1.53 (1.14–2.06)	1826/44	3.8 (2.8–5.1)	1.42 (0.97–2.07)	1122/29	4.1 (2.8–5.9)	1.72 (1.08–2.76)

Adjusted for age, sex, calendar year, smoking, hypertension, kidney function (eGFR), glucose-lowering drug therapy (including insulin), and HbA1c level. See Additional file 1: Table S1 for definitions of covariates

*CI* confidence interval; *CVD* cardiovascular disease; *HR* hazard ratio; *PY* person years; *T2DM*, type 2 diabetes mellitus

### Additional analyses

Further adjusting the main model for obesity, cancer, alcohol abuse, chronic liver disease, psychiatric comorbidity, and aspirin did not materially change the associations (Additional file 1: Table S3). Additional adjustment for LDL cholesterol attenuated the association between triglycerides and MACE slightly (adjusted HRs: triglycerides 1.0–1.9 mmol/L: 1.10 [95% CI 0.97–1.25]; triglycerides 2.0–2.9 mmol/L: 1.23 [95% CI 1.05–1.43]), except for triglycerides ≥ 3.0 mmol/L: 1.48 [95% CI 1.21–1.80]) (Additional file 1: Table S4). When follow-up was started 6 months after T2DM diagnosis based on early triglyceride measurements, adjusted HRs were similar to those obtained in the main analysis, but even higher for patients with triglyceride levels ≥ 3.0 mmol/L (HR of 1.56 [95% CI 1.28–1.90]) *vs.*1.44 [95% 1.20–1.73] in the main analysis) (Additional file 1: Table S5). Triglyceride levels above versus below 1.7 mmol/L yielded an adjusted HR of 1.26 (95% CI 1.15–1.38) for MACE while the adjusted HRs of guideline specific cut-off values (< 1.0 mmol/L, 1.0–1.69 mmol/L, 1.7–2.29 mmol/L, ≥ 2.3 mmol/L) were similar to those found in the main analysis (Additional file 1: Table S6). Patients treated with statins for > 365 days tended to have a slightly higher risk of MACE and each individual outcome, but no effect measure modification from statin treatment duration was observed overall (Additional file 1: Table S7). Compared with our study population (n = 27,080), statin users without a triglyceride measurement within one year after T2DM diagnosis (n = 4348) in general had similar baseline characteristics. However, they were more often diagnosed with T2DM before 2008 (26% of those not eligible vs. 16% of the study population) (Additional file 1: Table S8). Finally, the analysis of E-values showed that any unmeasured confounding factor associated with both triglycerides and MACE would have to be strong to account for our findings (Additional file 1: Table S9).

## Discussion

In this large cohort of 27,080 newly diagnosed T2DM patients treated with statins in routine clinical care, we observed that increasing triglyceride levels were associated with a gradually increasing risk of MACE, primarily driven by higher occurrence of myocardial infarction and cardiac death after 14 years of follow-up. This association was strongest among women and attenuated only slightly after further adjustment for LDL cholesterol. The novelty of these findings relates to a cohort of high-risk, statin-treated, newly diagnosed T2DM patients without a history of CVD events, i.e., at a time where the primary prevention effect may be most effective. Our findings thus demonstrate that triglycerides > 1.0 mmol/L within one year after T2DM diagnosis should be considered a predictor of residual CVD risk in newly diagnosed T2DM patients already treated with statin therapy and could be used to guide allocation of additional lipid-lowering therapies for CVD prevention [34].

In routine clinical care, LDL cholesterol is the primary lipid parameter used to monitor CVD risk [21, 22]. However, studies in the general population have shown that other triglyceride-rich lipoproteins are causally associated with CVD [2, 8, 35, 36]. Thus, although atherosclerosis is not primarily caused by elevated triglycerides [4, 37], triglyceride levels can be viewed as a marker of very low-density lipoprotein and intermediate-density lipoprotein remnants, which are not routinely measured in contemporary practice [1–4, 8, 36, 37].

The European Society of Cardiology and the American Diabetes Association recognize that triglyceride levels ≥ 1.7 mmol/L are associated with increased CVD risk, but recommend initiation of lipid-lowering add-on therapy only in high-risk patients with triglyceride levels > 2.3 mmol/L, after excluding other causes of hypertriglyceridemia (*e.g*., hyperglycemia) and encouraging lifestyle changes [21, 22]. In our population-based cohort of newly diagnosed T2DM patients, 22% had triglyceride levels > 2.3 mmol/L, despite current GLD and statin therapy [21, 22]. However, our spline models showed that the risk of MACE, myocardial infarction, stroke, and cardiac death increased rapidly with triglyceride levels between 1.0 and 2.0 mmol/L. American and European scientific statements have recommended that optimal triglyceride levels may be < 1.13 mmol/L [100 mg/dL] in the general population [2, 13]. Our data suggest that a lower triglyceride target for identifying patients at elevated risk of incident CVD should also be considered in newly diagnosed patients with T2DM already treated with statin therapy. This is important for interpreting triglyceride levels in current clinical practice.

Other studies have examined the association between elevated triglycerides and CVD risk among patients with longstanding T2DM [10, 14–16, 18, 19] although, as mentioned earlier, these cohorts did not address this relation in statin-treated high-risk patients with newly-diagnosed T2DM. A meta-analysis of 11 studies found that patients within the highest triglyceride category had a pooled adjusted risk ratio (RR) of 1.30 (95% CI 1.16–1.46) for a composite outcome of coronary heart disease and stroke, compared to those in the lowest triglyceride category [14]. In line with our results, the pooled RR was higher for females (RR 1.46 [95% CI 1.26–1.70]) than for males (RR 1.19 [95% CI 0.95–1.49]). Studies included in this meta-analysis were carried out in subgroups, i.e., men, ethnic minorities, and patients with a history of CVD. Further, many did not adjust for LDL cholesterol and/or lipid-lowering treatment, which hampered their ability to report residual risk of CVD beyond LDL cholesterol. Moreover, we reported the association using continuous spline models to examine a dose–response relationships, instead of comparing extreme contrasts when using categorial cut-offs [38].

A few previous studies, originating mainly from the US, have shown an association between elevated triglycerides and *specific* CVD events in cohorts *without known CVD* [15, 16, 19]. For example, data from 4199 T2DM patients in the Look Action for Health in Diabetes cohort showed that high triglyceride levels (≥ 1.7 mmol/L) associated with incident coronary artery disease defined as nonfatal myocardial infarction and/or coronary artery bypass grafting (adjusted HR of 1.16 [95% CI 0.95–1.42]), but not with ischemic and/or hemorrhagic stroke (0.97 [95% CI 0.65–1.45]) [15]. Using similar cut-offs, our study yielded slightly higher associations for myocardial infarction and a positive association for ischemic stroke. The associations of triglycerides with stroke strengthened when we initiated follow-up earlier, *i.e.*, at 6 months instead of the predefined 12 months, suggesting that stroke events often occurred close to the date of T2DM diagnosis. Other studies yielded comparable results to our findings, however, were not nested in routine clinical care and included patients with longstanding diabetes [16], prevalent CVD [10, 18], LDL cholesterol < 2.6 mmol/L [16, 18], or primarily native Americans [19]. Previous literature has also used patients with both normal HDL cholesterol and triglyceride values as the reference group [16, 19]. We focused only on triglycerides since HDL cholesterol is known to be strongly correlated with high triglyceride levels, yet not causally associated with CVD [2, 4, 22, 28, 29].

The U-shaped association of triglycerides and ischemic stroke is new and calls for a cautious interpretation. Previous studies of diabetes patients have either not examined the specific risk of ischemic stroke [10, 14, 16] or have not employed spline analyses that can reveal U-shaped associations [15, 18, 19]. One potential explanation may be the presence of underlying etiologies of ischemic stroke other than atherosclerosis in those with low triglycerides, e.g., atrial fibrillation. Our data suggest that a similar proportion of patients had atrial fibrillation across triglyceride levels. However, patients with triglycerides < 1.0 mmol/L tended to use anticoagulants less often, which could contribute to a higher risk of stroke caused by atrial fibrillation. However, further research on this topic is needed.

### Strengths and limitations

The strength and novelty of this study relate to a cohort of newly diagnosed, statin-treated T2DM patients without a history of CVD events. Thus, in contrast to previous literature, our study adds information on the impact of triglyceride levels in T2DM patients in routine clinical care early in the course of their disease where preventive initiatives may be most effective [21]. Other major strengths of this cohort study include its population-based design and its setting in a country with universal and free excess to health care, preventing selective inclusion of patients with specific health insurance systems and income levels [23]. Biomarker data in LABKA are of high quality due to standardized quality assurance measures implemented in Danish hospital laboratories [24].

Our study has limitations. First, using routine care registries and an age-based criterion to capture patients with T2DM, we may have misclassified some patients with late-onset type 1 diabetes, latent autoimmune diabetes of adults, or gestational diabetes as T2DM [25]. Second, despite adjustment for a wide range of confounders, such as obesity, smoking and alcohol abuse, the possible low sensitivity and the lack of details on these lifestyle markers in the Danish healthcare registries may have caused residual confounding [39]. Furthermore, we lacked information on physical activity. This may have caused an overestimation of the association between triglycerides and MACE. Still, our E-value analysis demonstrated that any unmeasured confounding factor would need to be very strong to explain away our findings. Third, due to the study’s population-based setting, not all patients had triglyceride measurements within one year after T2DM diagnosis, potentially hampering the generalizability of our results. However, our baseline characterization showed that statin users without a triglyceride measurement were very similar to our study population except for the fact that they were more often diagnosed with T2DM before 2008. The lack of triglyceride measurements may therefore be due to less complete reporting to the laboratory database and possibly less strict monitoring guidelines in the early years of the study period may have played a role in missing data [24], which supports that the results are generalizable to the full cohort of statin-treated T2DM patients without CVD within one year after diabetes diagnosis. Fourth, observed non-fasting triglyceride values may be influenced by regression towards the mean, *i.e.*, an unusually high triglyceride level likely will be lower at the next measurement [40]. To reduce this effect, we focused on the triglyceride level measured closest to one year after a T2DM diagnosis, when the initial effect of glucose- and lipid-lowering therapy likely already had played out. Moreover, we used non-fasting blood samples in which a small part of the measured triglyceride concentration represents non-atherosclerotic chylomicron particles. While we cannot preclude that the results would be different in fasting patients, using non-fasting blood samples may be more clinically relevant because the postprandial state represents an average atherogenic lipid profile during a 24-h period [8]. Fifth, despite the high PPV reported for myocardial infarction, and ischemic stroke in the Danish National Patient Register, the validity of cardiac death registered in the Danish Register of Causes of Death is more uncertain. A validation study from 2003 reported a PPV of 86% and a sensitivity of 63% for myocardial infarction as the cause of death [41]. Thus, misclassification may have occurred and caused an underestimation of the observed association, assuming that the outcome misclassification was independent of triglyceride levels [42].

## Conclusions

We found that elevated triglycerides are highly prevalent in statin-treated patients within one year after T2DM diagnosis. Elevated triglyceride levels are associated with gradually increasing risk of MACE already from triglyceride levels at 1.0 mmol/L. This suggests that high triglyceride levels are a marker of residual cardiovascular risk in early T2DM and could be used to guide allocation of additional lipid-lowering therapies for CVD prevention in routine clinical care.

## Supplementary Information

**Additional file 1: Table S1.** Information on registries used in the study and codes used to define exposures, outcomes, and baseline covariates. **Figure S1.** Study design and biomarker assessment window. **Methods S1.** Missing Values and Data Imputation. **Table S2.** Characteristics of 27,080 statin-treated patients according to their triglyceride level one year after T2DM diagnosis. **Table S3.** Adjusted hazard ratios of CVD events associated with triglyceride levels in statin-treated T2DM patients: Extensive adjustment. **Table S4.** Adjusted hazard ratios of CVD events associated with triglyceride levels in statin-treated T2DM patients: Additional adjustment for LDL cholesterol (N = 26,502). **Table S5.** Adjusted hazard ratios of CVD events associated with triglyceride levels in statin-treated T2DM patients: Triglycerides measured within 180 days *after* T2DM diagnosis (N = 19,980). **Table S6.** Adjusted hazard ratios of CVD events associated with the cut-off level for hypertriglyceridemia used in guidelines (≥1.7 mmol/L and ≥2.3 mmol/L). **Table S7.** Adjusted hazard ratios for study outcomes associated with triglyceride levels in statin-treated patients by statin duration. **Table S8.** Characteristics of statin patients with and without a triglyceride measurement within 365 days after T2DM diagnosis. **Table S9.** E-values for the association of MACE and secondary endpoints with triglyceride levels.

## Data Availability

The datasets supporting the conclusions of this article are available in the server of the Danish Health Data Authority (https://sundhedsdatastyrelsen.dk/da/english).

## Abbreviations:

T2DM: Type 2 diabetes mellitus
MACE: Major adverse cardiovascular events
HR: Hazard ratio
LDL: Low-density lipoprotein
CVD: Cardiovascular disease
LABKA: Clinical Laboratory Information System
GLD: Glucose-lowering drug
PPV: Positive predictive value
HDL: High-density lipoprotein
eGFR: Estimated glomerulus filtration rate
RR: Risk ratio
