# Supplementary material for: Triglycerides and risk of cardiovascular events in statin-treated patients with newly diagnosed type 2 diabetes: a Danish cohort study
Source: Cardiovasc Diabetol. 2023 Jul 26;22:187. doi: 10.1186/s12933-023-01921-5 (PMC10373341; doi:10.1186/s12933-023-01921-5)
Supplement: Supplementary file 1 — Additional file 1: Table S1. Information on registries used in the study and codes used to define exposures, outcomes, and baseline covariates. Figure S1. Study design and biomarker assessment window. Methods S1. Missing Values and Data Imputation. Table S2. Characteristics of 27,080 statin-treated patients according to their triglyceride level one year after T2DM diagnosis. Table S3. Adjusted hazard ratios of CVD events associated with triglyceride levels in statin-treated T2DM patients: Extensive adjustment. Table S4. Adjusted hazard ratios of CVD events associated with triglyceride levels in statin-treated T2DM patients: Additional adjustment for LDL cholesterol (N = 26,502). Table S5. Adjusted hazard ratios of CVD events associated with triglyceride levels in statin-treated T2DM patients: Triglycerides measured within 180 days after T2DM diagnosis (N = 19,980). Table S6. Adjusted hazard ratios of CVD events associated with the cut-off level for hypertriglyceridemia used in guidelines (≥1.7 mmol/L and ≥2.3 mmol/L). Table S7. Adjusted hazard ratios for study outcomes associated with triglyceride levels in statin-treated patients by statin duration. Table S8. Characteristics of statin patients with and without a triglyceride measurement within 365 days after T2DM diagnosis. Table S9. E-values for the association of MACE and secondary endpoints with triglyceride levels. [file 12933_2023_1921_MOESM1_ESM.docx]

Additional file

Title: Triglycerides and Risk of Cardiovascular Events in Statin-treated Patients with Newly Diagnosed Type 2 Diabetes: A Danish Cohort study

Authors: Frederik Pagh Bredahl Kristensen, MD, Diana Hedevang Christensen, MD, PhD, Martin Bødtker Mortensen, MD, PhD, Michael Maeng, MD, PhD, Johnny Kahlert, MSc, PhD, Henrik Toft Sørensen, MD, PhD, DSc, DMSc, Reimar Wernich Thomsen, MD, PhD.

| Description | Page |
| --- | --- |
| Table S1. Information on registries used in the study and codes used to define exposures, outcomes, and baseline covariates. | 2-6 |
| Figure S1. Study design and biomarker assessment window. | 7 |
| Methods S1 on Missing Values and Data Imputation. | 8 |
| Table S2. Characteristics of 27,080 statin-treated patients according to their triglyceride level one year after T2DM diagnosis. | 9-10 |
| Table S3. Adjusted hazard ratios of CVD events associated with triglyceride levels in statin-treated T2DM patients: **Extensive adjustment.** | 11 |
| Table S4. Adjusted hazard ratios of CVD events associated with triglyceride levels in statin-treated T2DM patients**: Additional adjustment for LDL cholesterol (N=26,502).** | 12 |
| Table S5. Adjusted hazard ratios of CVD events associated with triglyceride levels in statin-treated T2DM patients: **Triglycerides measured within 180 days *after* T2DM diagnosis** (N=19,980). | 13 |
| Table S6. Adjusted hazard ratios of CVD events associated with the cut-off level for hypertriglyceridemia used in **guidelines (≥1.7 mmol/L and ≥2.3 mmol/L)** | 14 |
| Table S7. Adjusted hazard ratios for study outcomes associated with triglyceride levels in statin-treated patients **by statin duration** | 15 |
| Table S8. Characteristics of statin patients with and without a triglyceride measurement within 365 days after T2DM diagnosis | 16 |
| Table S9. E-values for the association of MACE and secondary endpoints with triglyceride levels. | 17 |
| References | 18 |

**Table S1. Information on registries used in the study and codes used to define exposures, outcomes, and baseline covariates.**

| **Registry** | **Information** | |  |
| --- | --- | --- | --- |
| **Danish Civil Registration System** | Daily updated individual-level data on civil status, vital status, and migration since 1968 for the entire Danish population [1]. | | |
| **Danish National Patient Registry** | Nationwide hospital-related data, including diagnosis codes, surgical procedure codes, and discharge dates from inpatient hospitals in Denmark since 1977 and from outpatient clinics, emergency departments, and psychiatric facilities since 1995. Diagnosis codes have been recorded according to the *Eighth Revision* of the *International* *Classification of Diseases* (ICD-8) since 1977 and according to the *Tenth Revision* (ICD-10) since 1994 [2]. | | |
| **Danish National Prescription Registry** | Information on all reimbursed prescriptions redeemed at community pharmacies since 1995 [3]. | | |
| **Clinical Laboratory Information System Research Database (LABKA)** | Biochemistry data from routinely collected blood tests covering hospitals and general practitioners for one-third of the Danish population since 2005 [4]. | | |
| **Danish Register of Causes of Death** | Information on date and cause of deaths in Denmark since 1943. It is currently complete until the end of 2019. Causes of death have been registered according to ICD-10 since 1994. The database records information on the underlying cause of death, the immediate cause of death, contributory causes of death, and additional causes of death [5]. | | |
| **Variable** | **ICD-10 diagnosis codes, procedure codes, or surgical codes** | **ATC codes** | **Data sources (look-back period for covariates)** |
| **Diabetes definition[6]** | ICD-10: E10-E14, O24 [except O24.4], G63.2, H36.0, N08.3 | A10 | Danish National Patient Registry and the Danish National Prescription Registry |
| **Outcomes** |  |  |  |
| Myocardial infarction | Primary diagnosis of I21 associated with an inpatient hospitalization. Follow-up time until: April 30, 2021 |  | Danish National Patient Registry |
| Cardiac death | I20-I25, I46, I47.2, I49, I50, R96, R999, R989  Follow-up time until: December 31, 2019 |  | Danish Register of Causes of Death |
| Stroke overall | Primary or secondary diagnosis of I63 or I64 associated with an inpatient hospitalization. Follow-up time until: April 30, 2021 |  | Danish National Patient Registry |
| MACE | Occurrence of either myocardial infarction, stroke overall, or cardiac death (see codes above)  Follow-up time until: December 31, 2019 |  | Danish National Patient Registry and the Danish Register of Causes of Death |
| **Pre-existing cardiovascular diseases or related procedures (exclusion criteria).**  **All primary and secondary diagnosis codes related to all in- and outpatient hospital visits were included.** |  |  |  |
| Myocardial infarction | I21 |  | Danish National Patient Registry (until 1994) |
| Coronary revascularization (percutaneous coronary intervention/ coronary artery bypass grafting) | Surgical codes: KFNA, KFNB, KFNC, KFND, KFNE, KFNF, KFNG, KFNH, KFNW, KFLF |  | Danish National Patient Registry (until 1994) |
| Stroke overall | I63, I64 |  | Danish National Patient Registry (until 1994) |
| Peripheral artery disease | I70, I739, E105, E115, E125, E135, E145, I743 |  | Danish National Patient Registry (until 1994) |
| Angina pectoris | I20, I251, I259 |  | Danish National Patient Registry (until 1994) |
| Heart failure | I50, I110, I130, I132, I420, I426 I427, I428, I429 |  | Danish National Patient Registry (until 1994) |
| Hemorrhagic stroke | I60, I61, S066 |  | Danish National Patient Registry (until 1994) |
| Thrombolysis/thrombectomy after stroke | Surgical codes: KAAL10, KAAL11 |  | Danish National Patient Registry (until 1994) |
| Lower limb revascularization or lower limb amputation | Surgical codes: KPDE, KPDF, KPDH, KPDN, KPDP, KPDQ, KPEE, KPEF, KPEH, KPEN, KPEP, KPEQ, KPEU74, KPEU82, KPEU83, KPEU84, KPFE, KPFH, KPFN, KPFP, KPFQ, KPFU74, KPFU84, KNFQ, KNGQ, KNHQ |  | Danish National Patient Registry (until 1994) |
| **Comorbidities** |  |  |  |
| Smoking (binary variable) | Z587, Z720, J40-J44, J47 | R03BB, R03AC, R03AK, R03CC, R03DB, R03DA,  R03AL, R03BA | Danish National Patient Registry and the Danish National Prescription Registry (10-year look-back) |
| Atrial fibrillation (binary variable) | I48 |  | Danish National Patient Registry (10-year look-back) |
| Obesity (binary variable) | E65, E66, E68 |  | Danish National Patient Registry (10-year look-back) |
| Alcohol abuse (binary variable) | E244, E529, F10, G312, G621, G721, I426, K70, K852, K860, L278A, K292, R780, T51, Z714, Z721, I85, I864, I982 | N07BB | Danish National Patient Registry and the Danish National Prescription Registry (10-year look-back for diagnosis and 1-year look-back for medication) |
| Hypertension (either a hospital-recorded diagnosis of hypertension or use of at least *two* different classes of anti-hypertensive drugs prior to the index date) (binary variable) | I10-I15 | Antihypertensive drugs: α-adrenergic antihypertensive, non-loop diuretics and potassium-sparing agents, beta-blockers, ca-antagonists, renin-angiotensin-system antagonists (see ATC codes below). | Danish National Patient Registry and the Danish National Prescription Registry (10-year look-back for diagnosis and 1-year look-back for medication) |
| Diabetic eye complications (binary variable) | E103, E113, E123, E133, E143, H330, H332, H333, H334, H335, DH360, H340, H341, H342, H348, H349 H450, H360 H46, H540, H541, H542, H543, H544, H547, H268, DH281, H282, H269, H430, H431, H438 H439, I708A. Surgical codes: KCKC10, KCKC15, KCKD65 |  | Danish National Patient Registry (10-year look-back) |
| Diabetic kidney complications (binary variable) | E102, E112, DE122, E132, E142, I120, I131, I132, N083, N06, N17, N18, N19, R809, Z992. Procedure code: BJFD |  | Danish National Patient Registry (10-year look-back) |
| Chronic liver disease (binary variable) | B18, K72, K70, K71, K73, K74, K760, B150, B160, B162, B190, K766, I85 |  | Danish National Patient Registry (10-year look-back) |
| Cancer (binary variable) | C00–C99 |  | Danish National Patient Registry (10-year look-back) |
| **Drugs** | **ATC codes** | |  |
| Aspirin (binary variable) | B01AC06, N02BA01, N02BA51 | | Danish National Prescription Registry (1-year look-back) |
| Anticoagulants (binary variable) | B01AA03, B01AA04, B01AE07, B01AF01, B01AF02, B01AB04, B01AB10, B01AB05, B01AB01, B01AX05, B01AC04, B01AC24, B01AC22, B01AC07, B01AC25 | | Danish National Prescription Registry (1-year look-back) |
| Insulin (binary variable) | A10A | | Danish National Prescription Registry (1-year look-back) |
| Metformin (binary variable) | A10BA, A10BD (02, 03, 05, 07, 08, 10, 11, 13-18, 20, 22, 23, 25) | | Danish National Prescription Registry (1-year look-back) |
| Sulfonylureas (binary variable) | A10BB, A10BD01, A10BD04, A10BD02, A10BD06, A10BC01 | | Danish National Prescription Registry (1-year look-back) |
| DPP4-inihitors (binary variable) | A10BH, A10BD07, A10BD08, A10BD09, A10BD10, A10BD11, A10BD12, A10BD13, A10BD18, A10BD22, A10BD25 | | Danish National Prescription Registry (1-year look-back) |
| GLP-1-analogues (binary variable) | A10BX04, A10BJ01, A10BX07, A10BJ02, A10BX10, A10BJ03, A10BX13, A10BJ04, A10BX14, A10BJ05, A10BJ06, A10AE54, A10AE56 | | Danish National Prescription Registry (1-year look-back) |
| SGLT-2 inhibitors (binary variable) | A10BX09, A10BK01, A10BX11, A10BK02, A10BX12, A10BK03, A10BK04 A10BK05 A10BD15, A10BD16, A10BD19, A10BD20, A10BD21, A10BD23, A10BD24, A10BD25 | | Danish National Prescription Registry (1-year look-back) |
| Other anti-hyperglycemic drugs (binary variable) | A10BG, A10BD03, A10BD04, A10BD05, A10BD06, A10BD09, A10BD12, A10BD14, A10BD17, A10BX02, A10BX03, A10BX08, A10BF | | Danish National Prescription Registry (1-year look-back) |
| Use of glucose-lowering drugs (binary variable) | Non-insulin GLD monotherapy: One redeemed prescription of either metformin, sulfonylureas, DPP4-inhibitors, GLP-1-analouges, SGLT-2 inhibitors or other anti-hyperglycemic drugs in the year before the index date.  Insulin or GLD polytherapy: One redeemed prescription of insulin and/or at least one prescription for at least two different glucose-lowering drugs: metformin, sulfonylureas, DPP4-inhibitors, GLP-1-analouges, SGLT-2 inhibitors or other anti-hyperglycemic drugs in the year before the index date. | |  |
| Statins (binary variable) | C10AA, C10BA, C10BX | | Danish National Prescription Registry (1-year look-back) |
| Fibrates (binary variable) | C10AB | | Danish National Prescription Registry (1-year look-back) |
| Renin-angiotensin-system antagonists (binary variable) | C09 | | Danish National Prescription Registry (1-year look-back) |
| Ca-antagonists (binary variable) | C08 | | Danish National Prescription Registry (1-year look-back) |
| Beta-blockers (binary variable) | C07 | | Danish National Prescription Registry (1-year look-back) |
| α-adrenergic antihypertensives (binary variable) | C02A, C02B, C02C | | Danish National Prescription Registry (1-year look-back) |
| Loop diuretics (binary variable) | C03EB, C03C | | Danish National Prescription Registry (1-year look-back) |
| Non-loop diuretics and potassium-sparing agents (binary variable) | C02DA, C03A, C03B, C03D, C03EA. | | Danish National Prescription Registry (1-year look-back) |
| Antidepressants including SSRIs (binary variable) | N06A | | Danish National Prescription Registry (1-year look-back) |
| Antipsychotics and anticonvulsants (binary variable) | N05A, N03 | | Danish National Prescription Registry (1-year look-back) |
| **Laboratory tests** | **NPU and analysis codes** | |  |
| Triglycerides (continues variable) | NPU code: NPU03620, NPU04094  Analysis code: 3620, 4094, 1322442, 1422442, 1326052, 1426052, 110224, 1511285, 1622151, 1713620, 1813620 | | LABKA (1-year look-back) |
| HbA1c (continues variable) | NPU code: NPU27300, DNK35249, NPU03835, AAA00740, AAB00091, AAB00092.  Analysis codes: 1312154, 1312156, 1412154, 1412156, 153, 154, 159, 3835, 113835, 1510994, 1610135, 1712307, 1812307, 1512155, 110285, 114835, 1510994, 1510996, 1613835, 1712307, 1713835, 1817295 | | LABKA (1-year look-back) |
| Creatinine (continues variable) | NPU codes: NPU04998, NPU18016, NPU01807, ASS00356, ASS00354, ASS00355  Analysis codes: 110266, 1511235, 1511236, 1610154, 1611807, 1710301, 1711807,1811807, 1817156, 111016, 1311235, 1411235, 1511237, 1610296, 1710552, 1817428, 4998, 18016, 110959, 1311231, 1411231, 1511231.  If several creatinine measurements were available, the median value was calculated. eGFR was calculated using an algorithm recommended by the KDIGO: “Clinical Practice Guideline for the Evaluation and Management of Chronic Kidney Disease”. Kidney International Supplements (2013) 3, viii, January 2013. | | LABKA (1-year look-back) |
| Total cholesterol (continues variable) | NPU code: NPU01566  Analysis codes: 1566, 1511230, 110223, 1322441, 1326051, 1422441, 1426051, 1510981, 1622152, 1711566, 1811566 | | LABKA (1-year look-back) |
| LDL cholesterol (continues variable) | NPU codes: NPU01568, NPU10171, AAB00101, AAB00102  Analysis codes: 1514407, 1568, 10171, 110221, 1322444, 1326054, 1422444, 1426054, 1622154, 1711568, 1811568 | | LABKA (1-year look-back) |
| HDL cholesterol (continues variable) | NPU codes: NPU01567, NPU10157  Analysis codes: 1622153, 110222, 1567, 1514405, 1322443, 1422443, 1326053, 1426053, 1711567, 1811567 | | LABKA (1-year look-back) |

**Figure S1. Outline of the study design and biomarker assessment window.**

**Index date of start of follow-up^b^**

**First T2DM record^a^**

365-day assessment window

**Triglyceride assessment window**

**Pre-exposure period**

**Follow-up period**

**Statin therapy before latest triglyceride measurement^c^**

**Diagnosed cardiovascular disease^d^ before index date**

**Time**

^a^The first record of T2DM was defined as either 1) a first-time occurrence of a hospital inpatient or outpatient clinic diagnosis of diabetes or 2) a first-time redeemed prescription for a glucose-lowering drug (GLD) issued by either a primary care or a hospital-based physician.

^b^The follow-up period started one year after T2DM diagnosis. Patients were followed until MACE (a composite of myocardial infarction, ischemic stroke, and cardiac death), non-cardiac death, emigration, or end of the study period (December 31, 2019).

^c^Patients had redeemed a statin prescription within 1 year of their triglyceride measurement.

^d^Cardiovascular disease was defined based on previous hospital diagnoses of myocardial infarction, angina pectoris, heart failure, ischemic stroke, hemorrhagic stroke, or peripheral arterial disease; or previous PCI/CABG, thrombolysis/thrombectomy, or lower limb revascularization/amputation. Abbreviation: T2DM, type 2 diabetes mellitus

**Methods S1 on Missing values and Data Imputation**

Missingness of covariates varied from 1% to 3% (Supplementary Table 2). Missing values were most often seen for LDL cholesterol among patients with triglyceride levels >4 mmol/L, for which LDL cannot be calculated using the Friedewald equation. In a preliminary analysis, we found that missingness was independent of age, sex, and comorbidities (data not shown). This suggests that laboratory missingness is random and that the observed data can adequately predict missingness although it may be impossible to fully prove the ‘missing at random’ assumption [7].

We employed multiple imputation with chained equations to account for potential selection problems using a complete case analysis [7]. Linear regression was chosen as the underlying model since the incomplete covariates were continuously measured. Guided by the degree of missingness, 20 complete data sets were imputed with 10 iterations each [7, 8].

The imputation model included covariates that also were included in the adjustment models, as well as important auxiliary variables for enhancing prediction of a given covariate and its missingness. The auxiliary covariates were chosen based on clinical knowledge about the potential correlation between covariates.

We imputed missing values of total cholesterol, LDL and HDL cholesterol, creatinine, and HbA1c. Predictors of missingness with complete data included age, sex, calendar year, triglyceride levels, glucose-lowering drug treatment, hospital-diagnosed obesity, smoking (*i.e*., diagnosis or treatment for chronic obstructive pulmonary disease), hypertension (*i.e*., diagnosis codes or treatment), and the comorbidities and medications listed in Table 1 and Supplementary Table 2. We also included outcome indicators (both individually and MACE), the method for identifying diabetes patients (diagnosis codes *vs.* glucose-lowering drug treatment), time from diagnosed diabetes until triglyceride measurement, and the Nelson Aalen indicator to enhance prediction ability when using a Cox model [9].

Continuous variables with clearly non-normal (skewed) distributions were zero-skewness log-transformed, *i.e*., transformed to approximate normality before imputation. After running the imputation model, the values were transformed back to the original scale before analysis [8]. No convergence trend was seen in imputed values when plotting mean values and variances. The imputed models were validated by comparing the means, medians, and interquartile ranges of the first and last imputed dataset with those of the complete dataset. Rubin’s Rule was used when combining estimates from multiple imputed datasets [8].

| **Table S2. Characteristics of 27,080 statin-treated patients according to their triglyceride level one year after T2DM diagnosis.** | | | | | |
| --- | --- | --- | --- | --- | --- |
| **Triglyceride level (mmol/L)** | **<1.0** | **1.0-1.9** | **2.0-2.9** | **≥3.0** | **Total** |
| N | 4,563 (17%) | 14,124 (52%) | 5,445 (20%) | 2,948 (11%) | 27,080 (100%) |
| Male | 2,538 (56%) | 7,196 (51%) | 2,860 (53%) | 1,826 (62%) | 14,420 (53%) |
| Median age (quartiles) | 65 (56-72) | 64 (55-71) | 61 (52-69) | 56 (49-65) | 63 (54-70) |
| Age groups (years) |  |  |  |  |  |
| 30-39 | 92 (2%) | 305 (2%) | 204 (4%) | 179 (6%) | 780 (3%) |
| 40-49 | 450 (10%) | 1,436 (10%) | 795 (15%) | 617 (21%) | 3,298 (12%) |
| 50-59 | 1,008 (22%) | 3,420 (24%) | 1,471 (27%) | 956 (32%) | 6,855 (25%) |
| 60-69 | 1,565 (34%) | 4,801 (34%) | 1,729 (32%) | 775 (26%) | 8,870 (33%) |
| 70-79 | 1,120 (25%) | 3,254 (23%) | 967 (18%) | 334 (11%) | 5,675 (21%) |
| >79 | 328 (7%) | 908 (6%) | 279 (5%) | 87 (3%) | 1,602 (6%) |
| Calendar year |  |  |  |  |  |
| 2005-2008 | 789 (17%) | 2,379 (17%) | 824 (15%) | 459 (16%) | 4,451 (16%) |
| 2009-2012 | 1,955 (43%) | 5,574 (39%) | 1,970 (36%) | 961 (33%) | 10,460 (39%) |
| 2013-2016 | 1,341 (29%) | 4,191 (30%) | 1,743 (32%) | 971 (33%) | 8,246 (30%) |
| 2017-2018 | 478 (10%) | 1,980 (14%) | 908 (17%) | 557 (19%) | 3,923 (14%) |
| Years of follow-up for MACE (quartiles) | 8.2 (5.3-10.4) | 7.9 (4.7-10.4) | 7.4 (4.7-10.4) | 7.1 (4.2-10.0) | 7.8 (4.6-10.3) |
| Days from latest triglyceride measurement until start of follow-up one year after T2DM diagnosis (quartiles) | 91 (42-182) | 91 (40-183) | 87 (37-179) | 84 (36-176) | 89 (38-181) |
| Days from first statin initiation until start of follow-up one year after T2DM diagnosis (quartiles) | 365 (354-1,290) | 435 (359-1,640) | 547 (364-1,879) | 551 (362-1,947) | 435 (359-1,662) |
| New statin use (statins initiated <=1 year before latest triglyceride measurement) | 2,659 (58%) | 7,287 (52%) | 2,599 (48%) | 1,424 (48%) | 13,969 (52%) |
| Prevalent statin use (statins initiated >1 year before latest triglyceride measurement) | 1,904 (42%) | 6,837 (48%) | 2,846 (52) | 1,524 (52%) | 13,11 (48%) |
| Smoking | 444 (10%) | 1,474 (10%) | 660 (12%) | 342 (12%) | 2,920 (11%) |
| Alcohol abuse | 131 (3%) | 314 (2%) | 153 (3%) | 112 (4%) | 710 (3%) |
| Obesity | 220 (5%) | 1,193 (8%) | 633 (12%) | 320 (11%) | 2,366 (9%) |
| Hypertension | 1,928 (42%) | 6,635 (47%) | 2,636 (48%) | 1,347 (46%) | 12,546 (46%) |
| HbA1c (%) [mmol/mol] |  |  |  |  |  |
| ≤6.9 [52] | 3,879 (85%) | 11,458 (81%) | 4,044 (74%) | 1,888 (64%) | 21,269 (79%) |
| 7.0-7.9 [53-63] | 472 (10%) | 1,888 (13%) | 946 (17%) | 624 (21%) | 3,930 (15%) |
| ≥8.0 [64] | 154 (3%) | 630 (4%) | 396 (7%) | 404 (14%) | 1,584 (6%) |
| Missing | 58 (1%) | 148 (1%) | 59 (1%) | 32 (1%) | 297 (1%) |
| Total cholesterol (mmol/L) |  |  |  |  |  |
| <4.3 | 3,342 (73%) | 8,906 (63%) | 2,537 (47%) | 857 (29%) | 15,642 (58%) |
| ≥4.3 | 1,217 (27%) | 5,211 (37%) | 2,905 (53%) | 2,088 (71%) | 11,421 (42%) |
| Missing | <5 | <10 | <5 | <3 | 17 (0%) |
| LDL cholesterol (mmol/L) |  |  |  |  |  |
| <1.8 | 1,947 (43%) | 4,378 (31%) | 1,514 (28%) | 861 (29%) | 8,700 (32%) |
| 1.8.2.59 | 2,040 (45%) | 6,486 (46%) | 2,252 (41%) | 958 (32%) | 11,736 (43%) |
| ≥2.6 | 568 (12%) | 3,234 (23%) | 1,667 (31%) | 911 (31%) | 6,380 (24%) |
| Missing | 8 (0%) | 26 (0%) | 12 (0%) | 218 (7%) | 264 (1%) |
| HDL cholesterol (mmol/L) |  |  |  |  |  |
| >1.0/1.3 (M/F) | 3,819 (84%) | 8,907 (63%) | 2,330 (43%) | 728 (25%) | 15,784 (58%) |
| ≤1.0/1.3 (M/F) | 740 (16%) | 5,211 (37%) | 3,109 (57%) | 2,214 (75%) | 11,274 (42%) |
| Missing | <5 | <10 | <10 | <10 | 22 (0%) |
| Non-HDL cholesterol (mmol/L) |  |  |  |  |  |
| <2.7 | 3,730 (82%) | 7,955 (56%) | 1,533 (28%) | 316 (11%) | 13,534 (50%) |
| 2.7-3.4 | 644 (14%) | 4,070 (29%) | 2,026 (37%) | 795 (27%) | 7,535 (28%) |
| ≥3.5 | 185 (4%) | 2,091 (15%) | 1,880 (35%) | 1,829 (62%) | 5,985 (22%) |
| Missing | <5 | <10 | <10 | <10 | 26 (0%) |
| eGFR (mL/min/1.73m2) |  |  |  |  |  |
| ≤59 | 257 (6%) | 1,136 (8%) | 501 (9%) | 219 (7%) | 2,113 (8%) |
| 60-89 | 2,003 (44%) | 5,986 (42%) | 2,055 (38%) | 891 (30%) | 10,935 (40%) |
| ≥90 | 2,173 (48%) | 6,618 (47%) | 2,763 (51%) | 1,753 (59%) | 13,307 (49%) |
| Missing | 130 (3%) | 384 (3%) | 126 (2%) | 85 (3%) | 725 (3%) |
| Comorbidities |  |  |  |  |  |
| Diabetic eye complications | 160 (4%) | 480 (3%) | 142 (3%) | 76 (3%) | 858 (3%) |
| Diabetic kidney complications | 39 (1%) | 151 (1%) | 96 (2%) | 63 (2%) | 349 (1%) |
| Arterial fibrillation | 173 (4%) | 605 (4%) | 276 (5%) | 117 (4%) | 1,171 (4%) |
| Chronic liver disease | 49 (1%) | 144 (1%) | 64 (1%) | 44 (1%) | 301 (1%) |
| Any cancer | 315 (7%) | 1,048 (7%) | 399 (7%) | 181 (6%) | 1,943 (7%) |
| Identification of diabetes patients |  |  |  |  |  |
| GLD prescription | 3,811 (84%) | 12,206 (86%) | 4,702 (86%) | 2,536 (86%) | 23,255 (86%) |
| Diabetes diagnosis code | 752 (16%) | 1,918 (14%) | 743 (14%) | 412 (14%) | 3,825 (14%) |
| Medication use  Non-insulin GLD monotherapy | 3,789 (83%) | 11,829 (84%) | 4,411 (81%) | 2,222 (75%) | 22,251 (82%) |
| Insulin or GLD polytherapy | 774 (17%) | 2,295 (16%) | 1,034 (19%) | 726 (25%) | 4,829 (18%) |
| Insulin | 291 (6%) | 519 (4%) | 227 (4%) | 170 (6%) | 1,207 (4%) |
| Metformin | 3,769 (83%) | 12,291 (87%) | 4,837 (89%) | 2,622 (89%) | 23,519 (87%) |
| DPP4-inhibitors | 244 (5%) | 762 (5%) | 369 (7%) | 253 (9%) | 1,628 (6%) |
| Sulfonylureas | 600 (13%) | 1,744 (12%) | 642 (12%) | 419 (14%) | 3,405 (13%) |
| GLP-1 analogues | 24 (1%) | 188 (1%) | 110 (2%) | 75 (3%) | 397 (1%) |
| SGLT2-inhibitors | 18 (0%) | 117 (1%) | 79 (1%) | 59 (2%) | 273 (1%) |
| Other glucose-lowering drugs | 25 (1%) | 75 (1%) | 32 (1%) | 28 (1%) | 160 (1%) |
| Aspirin | 1,344 (29%) | 3,914 (28%) | 1,421 (26%) | 671 (23%) | 7,350 (27%) |
| Anticoagulants | 264 (6%) | 992 (7%) | 438 (8%) | 198 (7%) | 1,892 (7%) |
| Fibrates | 7 (0%) | 52 (0%) | 42 (1%) | 77 (3%) | 178 (1%) |
| Loop diuretics | 259 (6%) | 1,205 (9%) | 596 (11%) | 330 (11%) | 2,390 (9%) |
| Non-loop diuretics | 946 (21%) | 3,439 (24%) | 1,431 (26%) | 632 (21%) | 6,448 (24%) |
| Renin-angiotensin-system antagonists | 2,688 (59%) | 8,800 (62%) | 3,374 (62%) | 1,764 (60%) | 16,626 (61%) |
| Calcium channel antagonists | 1,191 (26%) | 4,035 (29%) | 1,456 (27%) | 775 (26%) | 7,457 (28%) |
| Beta blockers | 692 (15%) | 2,973 (21%) | 1,313 (24%) | 739 (25%) | 5,717 (21%) |
| Adrenergic antihypertensives | 44 (1%) | 161 (1%) | 61 (1%) | 44 (1%) | 310 (1%) |
| Antipsychotics and anticonvulsants | 250 (5%) | 1,065 (8%) | 616 (11%) | 418 (14%) | 2,349 (9%) |
| Antidepressants including SSRIs | 536 (12%) | 2,131 (15%) | 1,143 (21%) | 726 (25%) | 4,536 (17%) |

See Supplementary Table 1 for definitions of covariates.

Abbreviations: eGFR, glomerular filtration rate; GLD, glucose-lowering drug; HDL, high-density lipoprotein; LDL, low-density lipoprotein; SSRIs, selective serotonin reuptake inhibitors; T2DM, type 2 diabetes mellitus.

**Table S3. Adjusted hazard ratios of CVD events associated with triglyceride levels in statin-treated T2DM patients: Extensive adjustment.**

| Triglycerides (mmol/L) | N | Events | Incidence rates per 1000 PY (95% CI) | Main model adjusted HR (95% CI) | Extensively adjusted HR (95% CI) |
| --- | --- | --- | --- | --- | --- |
| MACE |  |  |  |  |  |
| <1.0 | 4,563 | 322 | 10.2 (9.2-11.4) | 1.0 | 1.0 |
| 1.0-1.9 | 14,124 | 1,051 | 11.2 (10.5-11.8) | 1.14 (1.00-1.29) | 1.13 (0.99-1.29) |
| 2.0-2.9 | 5,445 | 390 | 11.2 (10.3-12.5) | 1.30 (1.12-1.51) | 1.27 (1.09-1.48) |
| ≥3.0 | 2,948 | 194 | 10.6 (9.2-12.2) | 1.44 (1.20-1.73) | 1.38 (1.15-1.66) |
| Myocardial infarction | | |  |  |  |
| <1.0 | 4,563 | 84 | 2.3 (1.8-2.8) | 1.0 | 1.0 |
| 1.0-1.9 | 14,124 | 327 | 2.9 (2.6-3.3) | 1.35 (1.06-1.71) | 1.35 (1.06-1.72) |
| 2.0-2.9 | 5,445 | 141 | 3.5 (2.9-4.1) | 1.67 (1.27-2.20) | 1.68 (1.28-2.22) |
| ≥3.0 | 2,948 | 74 | 3.4 (2.7-4.3) | 1.75 (1.27-2.42) | 1.76 (1.27-2.44) |
| Ischemic stroke | | |  |  |  |
| <1.0 | 4,563 | 172 | 4.7 (4.0-5.4) | 1.0 | 1.0 |
| 1.0-1.9 | 14,124 | 483 | 4.4 (4.0-4.8) | 0.96 (0.81-1.15) | 0.97 (0.81-1.15) |
| 2.0-2.9 | 5,445 | 173 | 4.2 (3.7-4.9) | 1.04 (0.84-1.29) | 1.04 (0.84-1.29) |
| ≥3.0 | 2,948 | 88 | 4.0 (3.3-5.0) | 1.13 (0.86-1.47) | 1.12 (0.86-1.46) |
| Cardiac death | | |  |  |  |
| <1.0 | 4,563 | 134 | 4.1 (3.5-4.9) | 1.0 | 1.0 |
| 1.0-1.9 | 14,124 | 454 | 4.7 (4.3-5.2) | 1.20 (0.99-1.46) | 1.18 (0.97-1.43) |
| 2.0-2.9 | 5,445 | 161 | 4.6 (3.9-5.3) | 1.37 (1.08-1.72) | 1.29 (1.02-1.63) |
| ≥3.0 | 2,948 | 73 | 3.9 (3.1-4.9) | 1.53 (1.14-2.06) | 1.38 (1.03-1.85) |

The main model was adjusted for age, sex, calendar year, markers of smoking, hypertension, kidney function (eGFR), glucose-lowering drug therapy (including insulin), and HbA1c. The extensively adjusted model additionally included obesity, cancer, alcohol abuse, chronic liver disease, psychiatric comorbidity, and aspirin.

Abbreviations: PY, person years; HR, hazard ratio; CI, confidence interval; CVD, cardiovascular disease; T2DM, type 2 diabetes mellitus

**Table S4. Adjusted hazard ratios of CVD events associated with triglyceride levels in statin-treated T2DM patients: Additional adjustment for LDL cholesterol (N=26,502).**

| Triglycerides (mmol/L) | N | Events | Rates per 1000 PY (95% CI) | Main model adjusted HR (95% CI) | Main model and LDL cholesterol-adjusted HR (95% CI) |
| --- | --- | --- | --- | --- | --- |
| MACE |  |  |  |  |  |
| <1.0 | 4,567 | 322 | 10.2 (9.1-11.4) | 1.0 | 1.0 |
| 1.0-1.9 | 14,173 | 1.051 | 11.1 (10.5-11.8) | 1.14 (1.00-1.29) | 1.10 (0.97-1.25 |
| 2.0-2.9 | 5,630 | 397 | 11.2 (10.1-12.3) | 1.30 (1.12-1.51) | 1.23 (1.05-1.43) |
| ≥3.0 | 2,132 | 154 | 11.6 (9.9-13.6) | 1.57 (1.29-1.91) | 1.48 (1.21-1.80) |
| Myocardial infarction | | |  |  |  |
| <1.0 | 4,567 | 84 | 2.3 (1.8-2.8) | 1.0 | 1.0 |
| 1.0-1.9 | 14,173 | 327 | 2,9 (2.6-3.3) | 1.35 (1.06-1.71) | 1.22 (0.96-1.56) |
| 2.0-2.9 | 5,630 | 142 | 3.4 (2.9-4.0) | 1.65 (1.26-2.17) | 1.41 (1.07-1.87) |
| ≥3.0 | 2,132 | 60 | 3.8 (2.90-4.9) | 1.98 (1.41-2.78) | 1.69 (1.20-2.38) |
| Ischemic stroke | | |  |  |  |
| <1.0 | 4,567 | 172 | 4.7 (4.0-5.4) | 1.0 | 1.0 |
| 1.0-1.9 | 14,173 | 483 | 4.4 (4.0-4.8) | 0.96 (0.81-1.15) | 0.93 (0.78-1.11) |
| 2.0-2.9 | 5,630 | 175 | 4.1 (3.6-4.8) | 1.03 (0.83-1.27) | 0.97 (0.78-1.21) |
| ≥3.0 | 2,132 | 69 | 4.4 (3.4-5.5) | 1.21 (0.91-1.61) | 1.15 (0.86-1.53) |
| Cardiac death | | |  |  |  |
| <1.0 | 4,567 | 134 | 4.2 (3.5-4.9) | 1.0 | 1.0 |
| 1.0-1.9 | 14,173 | 454 | 4.7 (4.3-5.2) | 1.20 (0.99-1.46) | 1.20 (0.99-1.46) |
| 2.0-2.9 | 5,630 | 165 | 4.5 (3.9-5.3) | 1.38 (1.09-1.74) | 1.37 (1.09-1.74) |
| ≥3.0 | 2,132 | 58 | 4.3 (3.3-5.5) | 1.64 (1.20-2.26) | 1.64 (1.19-2.25) |

The cohort was restricted to patients with triglyceride levels ≤4.0 mmol/L, as LDL cholesterol at higher levels cannot be validly estimated using Friedewald’s equation [10, 11]. After excluding n=578 (2.1%) patients with triglyceride levels ≥ 4.0 mmol/L, 59 persons (0.2%) had missing LDL cholesterol levels. These levels were imputed (See “Information on Missing Values and Data Imputation” above).

The main model was adjusted for age, sex, calendar year, markers of smoking, hypertension, kidney function (eGFR), glucose-lowering drug therapy (including insulin), and HbA1c.
Abbreviations: PY, person years; HR, hazard ratio; CI, confidence interval; CVD, cardiovascular disease; LDL, low-density lipoprotein; T2DM, type 2 diabetes mellitus

**Table S5. Adjusted hazard ratios of CVD events associated with triglyceride levels in statin-treated T2DM patients: Triglycerides measured within 180 days *after* T2DM diagnosis.**

| Triglyceride levels (mmol/L) | N | Events | Rates per 1000 PY (95% CI) | Adjusted HR (95% CI) |
| --- | --- | --- | --- | --- |
| MACE |  |  |  |  |
| <1.0 | 3,220 | 260 | 10.9 (9.7-12.3) | 1.0 |
| 1.0-1.9 | 10,636 | 832 | 10.9 (10.2-11.7) | 1.09 (0.95-1.25) |
| 2.0-2.9 | 3,934 | 299 | 11.2 (10.0-12.6) | 1.28 (1.08-1.26) |
| ≥3.0 | 2,190 | 173 | 11.8 (10.2-13.7) | 1.56 (1.28-1.90) |
| Myocardial infarction | | |  |  |
| <1.0 | 3,220 | 73 | 2.6 (2.1-3.3) | 1.0 |
| 1.0-1.9 | 10,636 | 258 | 2.9 (2.6-3.3) | 1.17 (0.90-1.52) |
| 2.0-2.9 | 3,934 | 101 | 3.2 (2.7-3.9) | 1.40 (1.03-1.90) |
| ≥3.0 | 2,190 | 58 | 3.3 (2.6-4.3) | 1.50 (1.04-2.14) |
| Ischemic stroke | | |  |  |
| <1.0 | 3,220 | 148 | 5.4 (4.6-6.3) | 1.0 |
| 1.0-1.9 | 10,636 | 413 | 4.7 (4.2-5.2) | 0.94 (0.78-1.13) |
| 2.0-2.9 | 3,934 | 147 | 4.7 (4.0-5.5) | 1.07 (0.85-1.35) |
| ≥3.0 | 2,190 | 90 | 5.2 (4.2-6.4) | 1.34 (1.02-1.76) |
| Cardiac death | | |  |  |
| <1.0 | 3,220 | 102 | 4.2 (3.4-5.1) | 1.0 |
| 1.0-1.9 | 10,636 | 345 | 4.4 (4.0-4.9) | 1.18 (0.95-1.48) |
| 2.0-2.9 | 3,934 | 115 | 4.2 (3.5-5.0) | 1.33 (1.01-1.75) |
| ≥3.0 | 2,190 | 66 | 4.4 (3.4-5.6) | 1.72 (1.25-2.37) |

The model was adjusted for age, sex, calendar year, markers of smoking, hypertension, kidney function (eGFR), glucose-lowering drug therapy (including insulin), and HbA1c.

Abbreviations: PY, person years; HR, hazard ratio; CI, confidence interval; CVD, cardiovascular disease; T2DM, type 2 diabetes mellitus

**Table S6. Adjusted hazard ratios of CVD events associated with the cut-off level for hypertriglyceridemia used in guidelines (≥1.7 mmol/L and ≥2.3 mmol/L)**

| Triglycerides (mmol/L) | N | Events | Main model adjusted HR (95% CI) | |
| --- | --- | --- | --- | --- |
| MACE |  |  |  | |
| <1.7 | 15,412 | 1,107 | 1.0 | |
| ≥1.7 | 11,668 | 850 | 1.26 (1.15-1.38) | |
| Myocardial infarction | | | |  |
| <1.7 | 15,412 | 329 | 1.0 | |
| ≥1.7 | 11,668 | 297 | 1.35 (1.15-1.59) | |
| Ischemic stroke | | | |  |
| <1.7 | 15,412 | 525 | 1.0 | |
| ≥1.7 | 11,668 | 391 | 1.18 (1.03-1.35) | |
| Cardiac death | | | |  |
| <1.7 | 15,412 | 481 | 1.0 | |
| ≥1.7 | 11,668 | 341 | 1.23 (1.06-1.41) | |
| MACE |  |  |  | |
| <1.0 | 4,563 | 322 | 1.0 | |
| 1.0-1.9 | 10,849 | 785 | 1.09 (0.95-1.24) | |
| 2.0-2.9 | 5,605 | 449 | 1.36 (1.18-1.58) | |
| ≥3.0 | 6,063 | 401 | 1.31 (1.12-1.52) | |
| Myocardial infarction |  |  |  | |
| <1.0 | 4,563 | 84 | 1.0 | |
| 1.0-1.9 | 10,849 | 245 | 1.30 (1.01-1.67) | |
| 2.0-2.9 | 5,605 | 147 | 1.62 (1.24 (1.24-2.13) | |
| ≥3.0 | 6,063 | 150 | 1.66 (1.26-2.18) | |
| Ischemic stroke |  |  |  | |
| <1.0 | 4,563 | 172 | 1.0 | |
| 1.0-1.9 | 10,849 | 353 | 0.90 (0.75-1.09) | |
| 2.0-2.9 | 5,605 | 208 | 1.14 (0.93-1.40) | |
| ≥3.0 | 6,063 | 183 | 1.06 (0.85-1.31) | |
| Cardiac death |  |  |  | |
| <1.0 | 4,563 | 134 | 1.0 | |
| 1.0-1.9 | 10,849 | 347 | 1.17 (0.96-1.43) | |
| 2.0-2.9 | 5,605 | 181 | 1.37 (1.09-1.71) | |
| ≥3.0 | 6,063 | 160 | 1.38 (1.09-1.75) | |

The cut-off levels for hypertriglyceridemia were ascertained from the European Society of Cardiology and the American Diabetes Association guidelines [12, 13]. The main model was adjusted for age, sex, calendar year, markers of smoking, hypertension, kidney function (eGFR), glucose-lowering drug therapy (including insulin), and HbA1c.

Abbreviations: PY, person years; HR, hazard ratio; CI, confidence interval; CVD, cardiovascular disease

|  | Overall | | Statin duration ≤365 days | | Statin duration >365 days | |
| --- | --- | --- | --- | --- | --- | --- |
| Triglyceride level (mmol/L) | **N/events** | **Adjusted HR (95% CI)** | **N/events** | **Adjusted HR (95% CI)** | **N/events** | **Adjusted HR (95% CI)** |
| Mace |  |  |  |  |  |  |
| <1.0 | 4,563/322 | 1.0 | 2,339/159 | 1.0 | 2,224/163 | 1.0 |
| 1.0-1.9 | 14,124/1,051 | 1.14 (1.00-1.29) | 6,474/466 | 1.13 (0.94-1.36) | 7,650/585 | 1.16 (0.98-1.39) |
| 2.0-2.9 | 5,445/390 | 1.30 (1.12-1.51) | 2,314/145 | 1.19 (0.95-1.50) | 3,131/245 | 1.42 (1.16-1.74) |
| ≥3.0 | 2,948/194 | 1.44 (1.20-1.73) | 1,274/72 | 1.32 (0.98-1.76) | 1,674/122 | 1.61 (1.26-2.05) |
| Myocardial infarction | |  |  |  |  |  |
| <1.0 | 4,563/84 | 1.0 | 2,339/38 | 1.0 | 2,224/46 | 1.0 |
| 1.0-1.9 | 14,124/327 | 1.35 (1.06-1.71) | 6,474/142 | 1.40 (0.98-2.02) | 7,650/185 | 1.30 (0.94-1.80) |
| 2.0-2.9 | 5,445/141 | 1.67 (1.27-2.20) | 2,314/52 | 1.61 (1.06-2.47) | 3,131/89 | 1.70 (1.18-2.44) |
| ≥3.0 | 2,948/74 | 1.75 (1.27-2.42) | 1,274/27 | 1.62 (0.97-2.71) | 1,674/47 | 1.81 (1.19-2.76) |
| Ischemic stroke |  |  |  |  |  |  |
| <1.0 | 4,563/172 | 1.0 | 2,339/81 | 1.0 | 2,224/91 | 1.0 |
| 1.0-1.9 | 14,124/483 | 0.96 (0.81-1.15) | 6,474/211 | 0.98 (0.76-1.27) | 7,650/272 | 0.95 (0.75-1.21) |
| 2.0-2.9 | 5,445/173 | 1.04 (0.84-1.29) | 2,314/67 | 1.05 (0.75-1.46) | 3,131/106 | 1.05 (0.79-1.40) |
| ≥3.0 | 2,948/88 | 1.13 (0.86-1.47) | 1,274/33 | 1.08 (0.71-1.54) | 1,674/55 | 1.20 (0.85-1.69) |
| Cardiac death |  |  |  |  |  |  |
| <1.0 | 4,563/134 | 1.0 | 2,339/71 | 1.0 | 2,224/63 | 1.0 |
| 1.0-1.9 | 14,124/454 | 1.20 (0.99-1.46) | 6,474/204 | 1.14 (0.87-1.50) | 7,650/250 | 1.30 (0.98-1.72) |
| 2.0-2.9 | 5,445/161 | 1.37 (1.08-1.72) | 2,314/67 | 1.32 (0.94-1.86) | 3,131/94 | 1.51 (1.09-2.09) |
| ≥3.0 | 2,948/73 | 1.53 (1.14-2.06) | 1,274/31 | 1.55 (1.00-2.41) | 1,674/42 | 1.71 (1.14-2.56) |

**Table S7. Adjusted hazard ratios for study outcomes associated with triglyceride levels in statin-treated patients by statin duration**

The model was adjusted for age, sex, calendar year, markers of smoking, hypertension, kidney function (eGFR), glucose-lowering drug therapy (including insulin), and HbA1c.

Abbreviations: PY, person years; HR, hazard ratio; CI, confidence interval.

**Table S8. Characteristics of statin patients with and without a triglyceride measurement within 365 days after T2DM diagnosis.**

|  | **Statin users without a triglyceride measurement within 365 days after T2DM diagnosis** | **Statin users with a triglyceride measurement within 365 days after T2DM diagnosis (study population)** |
| --- | --- | --- |
| N | N=4,348 | N=27,082 |
| Males | 2,370 (55%) | 14,421 (53%) |
| Median age (quartiles) | 64 (55-72) | 63 (54-70) |
| Age groups (years) |  |  |
| 30-39 | 110 (3%) | 780 (3%) |
| 40-49 | 478 (11%) | 3,298 (12%) |
| 50-59 | 992 (23%) | 6,856 (25%) |
| 60-69 | 1,396 (32%) | 8,871 (33%) |
| 70-79 | 982 (23%) | 5,675 (21%) |
| >79 | 390 (9%) | 1,602 (6%) |
| Calendar year |  |  |
| 2005-2008 | 1,142 (26%) | 4,452 (16%) |
| 2009-2012 | 1,642 (38%) | 10,461 (39%) |
| 2013-2016 | 1,110 (26%) | 8,246 (30%) |
| 2017-2018 | 454 (10%) | 3,923 (14%) |
| Obesity | 369 (8%) | 2,366 (9%) |
| Smoking | 212 (5%) | 2,920 (11%) |
| Alcohol abuse | 110 (3%) | 710 (3%) |
| Hypertension | 2,160 (50%) | 12,547 (46%) |
| HbA1c (%) [mmol/mol], n | 2,664 (39% = missing) | 26,785 (1% = missing) |
| ≤6.9 [52] | 2,065 (76%) | 21,270 (79%) |
| 7.0-7.9 [53-63] | 423 (16%) | 3,930 (15%) |
| ≥8.0 [64] | 176 (7%) | 1,585 (6%) |
| Diabetic eye complications | 127 (3%) | 858 (3%) |
| Diabetic kidney complications | 68 (2%) | 349 (1%) |
| Arterial fibrillation | 197 (5%) | 463 (2%) |
| Chronic liver disease | 45 (1%) | 301 (1%) |
| Any cancer | 433 (10%) | 1,943 (7%) |
| Number of patients by definition of diabetes |  |  |
| GLD prescription | 3,785 (87%) | 23,257 (86%) |
| Diabetes diagnosis code | 563 (13%) | 3,825 (14%) |
| Intensity of GLD treatment |  |  |
| Monotherapy | 3,654 (84%) | 22,251 (82%) |
| Insulin or GLD polytherapy | 694 (16%) | 4,829 (18%) |
| Metformin | 3,527 (81%) | 23,521 (87%) |
| Insulin | 196 (5%) | 1,207 (4%) |
| Aspirin | 1,344 (31%) | 7,350 (27%) |
| Anticoagulants | 340 (8%) | 1,892 (7%) |
| Statins | 4,348 (100%) | 27,082 (100%) |
| Loop diuretics | 479 (11%) | 2,391 (9%) |
| Non-loop diuretics | 1,167 (27%) | 6,448 (24%) |
| Renin-angiotensin-system antagonists | 2,700 (62%) | 16,628 (61%) |
| Calcium channel antagonists | 1,239 (28%) | 7,457 (28%) |
| Beta blockers | 1,071 (25%) | 5,717 (21%) |

See Supplementary Table 1 for definitions of covariates.

Abbreviations: eGFR, glomerular filtration rate; GLD, glucose-lowering drug; HDL, high-density lipoprotein; LDL, low-density lipoprotein; SSRIs, selective serotonin reuptake inhibitors; T2DM, type 2 diabetes mellitus

**Table S9. E-values for the association of MACE and secondary endpoints with triglyceride levels.**

| Triglyceride levels (mmol/L) | N | Events | Rates per 1000 PY (95% CI) | | Main model adjusted HR (95% CI) | | E-value of point estimate | |
| --- | --- | --- | --- | --- | --- | --- | --- | --- |
| MACE | | | |  | |  | |  |
| <1.0 | 4,563 | 322 | 10.2 (9.2-11.4) | | 1.0 | |  | |
| 1.0-1.9 | 14,124 | 1,051 | 11.2 (10.5-11.8) | | 1.14 (1.00-1.29) | | 1.42 | |
| 2.0-2.9 | 5,445 | 390 | 11.3 (10.3-12.5) | | 1.30 (1.12-1.51) | | 1.69 | |
| ≥3.0 | 2,948 | 194 | 10.6 (9.2-12.2) | | 1.44 (1.20-1.73) | | 1.90 | |
| Myocardial infarction | | | |  | |  | |  |
| <1.0 | 4,563 | 84 | 2.3 (1.8-2.8) | | 1.0 | |  | |
| 1.0-1.9 | 14,124 | 327 | 2.9 (2.6-3.3) | | 1.35 (1.06-1.71) | | 1.76 | |
| 2.0-2.9 | 5,445 | 141 | 3.5 (2.9-4.1) | | 1.67 (1.27-2.20) | | 2.20 | |
| ≥3.0 | 2,948 | 74 | 3.4 (2.7-4.3) | | 1.75 (1.27-2.42) | | 2.31 | |
| Ischemic stroke | | | |  | |  | |  |
| <1.0 | 4,563 | 172 | 4.7 (4.0-5.4) | | 1.0 | |  | |
| 1.0-1.9 | 14,124 | 483 | 4.4 (4.0-4.8) | | 0.96 (0.81-1.15) | | 1.20 | |
| 2.0-2.9 | 5,445 | 173 | 4.2 (3.7-4.9) | | 1.04 (0.84-1.29) | | 1.20 | |
| ≥3.0 | 2,948 | 88 | 4.0 (3.3-5.0) | | 1.13 (0.86-1.47) | | 1.40 | |
| Cardiac death | | | |  | |  | |  |
| <1.0 | 4,563 | 134 | 4.1 (3.5-4.9) | | 1.0 | |  | |
| 1.0-1.9 | 14,124 | 454 | 4.7 (4.3-5.2) | | 1.20 (0.99-1.46) | | 1.53 | |
| 2.0-2.9 | 5,445 | 161 | 4.6 (3.9-5.3) | | 1.37 (1.08-1.72) | | 1.79 | |
| ≥3.0 | 2,948 | 73 | 3.9 (3.1-4.9) | | 1.53 (1.14-2.06) | | 2.02 | |

The main model was adjusted for age, sex, calendar year, markers of smoking, hypertension, kidney function (eGFR), glucose-lowering drug therapy (including insulin), and HbA1c. See Supplementary Table 1 for definitions of covariates.

Abbreviations: HR, hazard ratio; CI, confidence interval

**E-values:**

The E-value is defined as the minimum strength of association (risk ratio) that an unmeasured confounder would need to have with both the exposure and the outcome, to fully explain the given observed association (adjusted HR). A large E-value suggests that strong unmeasured confounding would be needed to explain the association, while a small E-value suggests that little unmeasured confounding would be needed [14]. **Example:** the exposure-confounder association and the confounder-outcome association would have to show a risk ratio of 1.9 or higher to explain away the observed association of triglycerides with MACE of (adjusted HR 1.44, comparing triglyceride levels <1.0 mmol/L with triglycerides levels ≥3.0 mmol/L). E-values were computed in Stata using the package “evalue” based on the methodology proposed by VanderWeele and Ding (2017) [14, 15].

**References**

1. Schmidt M, Pedersen L, Sorensen HT. The Danish Civil Registration System as a tool in epidemiology. Eur J Epidemiol. 2014 Aug;29(8):541-549.

2. Schmidt M, Schmidt SA, Sandegaard JL, Ehrenstein V, Pedersen L, Sorensen HT. The Danish National Patient Registry: a review of content, data quality, and research potential. Clin Epidemiol. 2015;7:449-490

3. Pottegard A, Schmidt SAJ, Wallach-Kildemoes H, Sorensen HT, Hallas J, Schmidt M. Data Resource Profile: The Danish National Prescription Registry. Int J Epidemiol. 2017;46(3):798-f.

4. Arendt JFH, Hansen AT, Ladefoged SA, Sørensen HT, Pedersen L, Adelborg K. Existing Data Sources in Clinical Epidemiology: Laboratory Information System Databases in Denmark. Clin Epidemiol. 2020;12:469-75.

5. Helweg-Larsen K. The Danish Register of Causes of Death. Scand. J. Public Health. 2011;39(7 Suppl):26-29.

6. Carstensen B, Rønn PF, Jørgensen ME. Prevalence, incidence and mortality of type 1 and type 2 diabetes in Denmark 1996-2016. BMJ Open Diabetes Res Care. 2020;8(1).

7. Sterne JA, White IR, Carlin JB, Spratt M, Royston P, Kenward MG, et al. Multiple imputation for missing data in epidemiological and clinical research: potential and pitfalls. Bmj. 2009;338:b2393.

8. White IR, Royston P, Wood AM. Multiple imputation using chained equations: Issues and guidance for practice. Stat med 2011;30(4):377-399.

9. White IR, Royston P. Imputing missing covariate values for the Cox model. Stat med. 2009;28(15):1982-98.

10. Friedewald WT, Levy RI, Fredrickson DS. Estimation of the concentration of low-density lipoprotein cholesterol in plasma, without use of the preparative ultracentrifuge. Clin Chem. 1972;18(6):499-502.

11. Martin SS, Blaha MJ, Elshazly MB, Toth PP, Kwiterovich PO, Blumenthal RS, et al. Comparison of a novel method vs the Friedewald equation for estimating low-density lipoprotein cholesterol levels from the standard lipid profile. JAMA. 2013;310(19):2061-8.

12. Visseren FLJ, Mach F, Smulders YM, Carballo D, Koskinas KC, Bäck M, et al. 2021 ESC Guidelines on cardiovascular disease prevention in clinical practice. Eur Heart H J. 2021;42(34):3227-337.

13. American Diabetes Association Professional Practice Committee. Standards of Medical Care in Diabetes-2022. Diabetes Care. 2022;45(Suppl 1):S1-S259.

14. VanderWeele TJ, Ding P. Sensitivity Analysis in Observational Research: Introducing the E-Value. Ann Intern Med. 2017;167(4):268-274.

15. Linden A, Mathur MB, VanderWeele TJ. EVALUE: Stata module for conducting sensitivity analyses for unmeasured confounding in observational studies. Statistical Software Components. Boston College Department of Economics2019.
